# Supplementary material for: Aquatic Toxicity Effects and Risk Assessment of ‘Form Specific’ Product-Released Engineered Nanomaterials
Source: Int J Mol Sci. 2021 Nov 18;22(22):12468. doi: 10.3390/ijms222212468 (PMC8621863; doi:10.3390/ijms222212468)
Supplement: Supplementary file 1 [file ijms-22-12468-s001.zip › ijms-1438619-supplementary.pdf]

Article

Aquatic Toxicity Effects and Risk Assessment of 'Form Specific' Product-Released Engineered Nanomaterials

Raisibe Florence Lehutso [1,2], James Wesley-Smith [3] and Melusi Thwala [1,4,*]

[1] Water Centre, Council for Scientific and Industrial Research, Pretoria 0001, South Africa
[2] Department of Chemical Sciences, University of Johannesburg, Johannesburg 2028, South Africa
[3] Electron Microscope Unit, Sefako Makgatho Health Sciences University, Pretoria 0001, South Africa
[4] Centre for Environmental Management, University of the Free State, Bloemfontein 9031, South Africa
* Correspondence: mthwala@csir.co.za

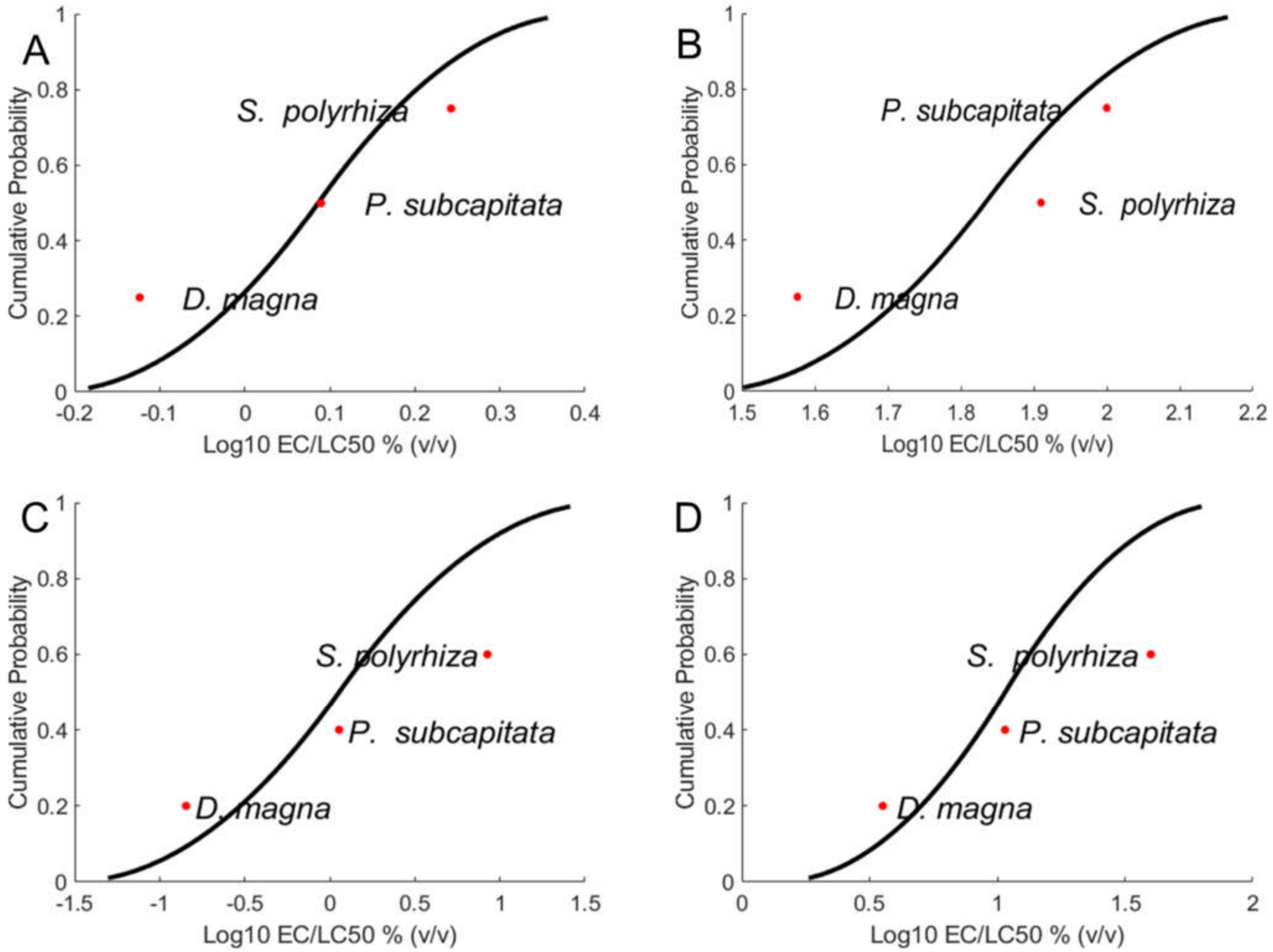

Figure S1. Species sensitivity distributions plot illustrating different responses of the three aquatic organisms to SUN1 (**A**), CA1 (**B**) SAN1 ions; in all cases, *D. magna* was the most sensitive organism.

1.1. S. Evaluation of binary PR-ENMs

SUN1 PR-nTiO_2 and PR-nZnO mainly induced synergistic or antagonistic effects in the three test organisms (Figures 2A–C). The observed effects of PR-nTiO_2 and PR-nZnO and the predicted effects were significantly different. Few exceptions where PR-nZnO-induced additive effects were recorded. PR-nZnO obtained under dark conditions induced additive effects on *S. polyrhiza*. Additive effects were also induced on *D. magna* at 24 h and 48 h by PR-nZnO obtained under light and dark conditions, respectively. As the analyte responsible for causing either synergistic or antagonistic effects could not be established, the LC/EC_{50} expression relative to the total concentration of an analyte was considered impossible for SUN1 and therefore was not determined. CA1 and SK1 PR-nTiO_2 and PR-nAg induced either synergistic or antagonistic effects in all three test organisms in all test scenarios (Figures S2A–C). The observed effects [P(O)] and theoretical effects [P(E)] were different. Similarly, to SUN1 PR–ENMs, the LC/EC_{50} expression relative to an analyte's total concentration was considered impossible and thus not determined.

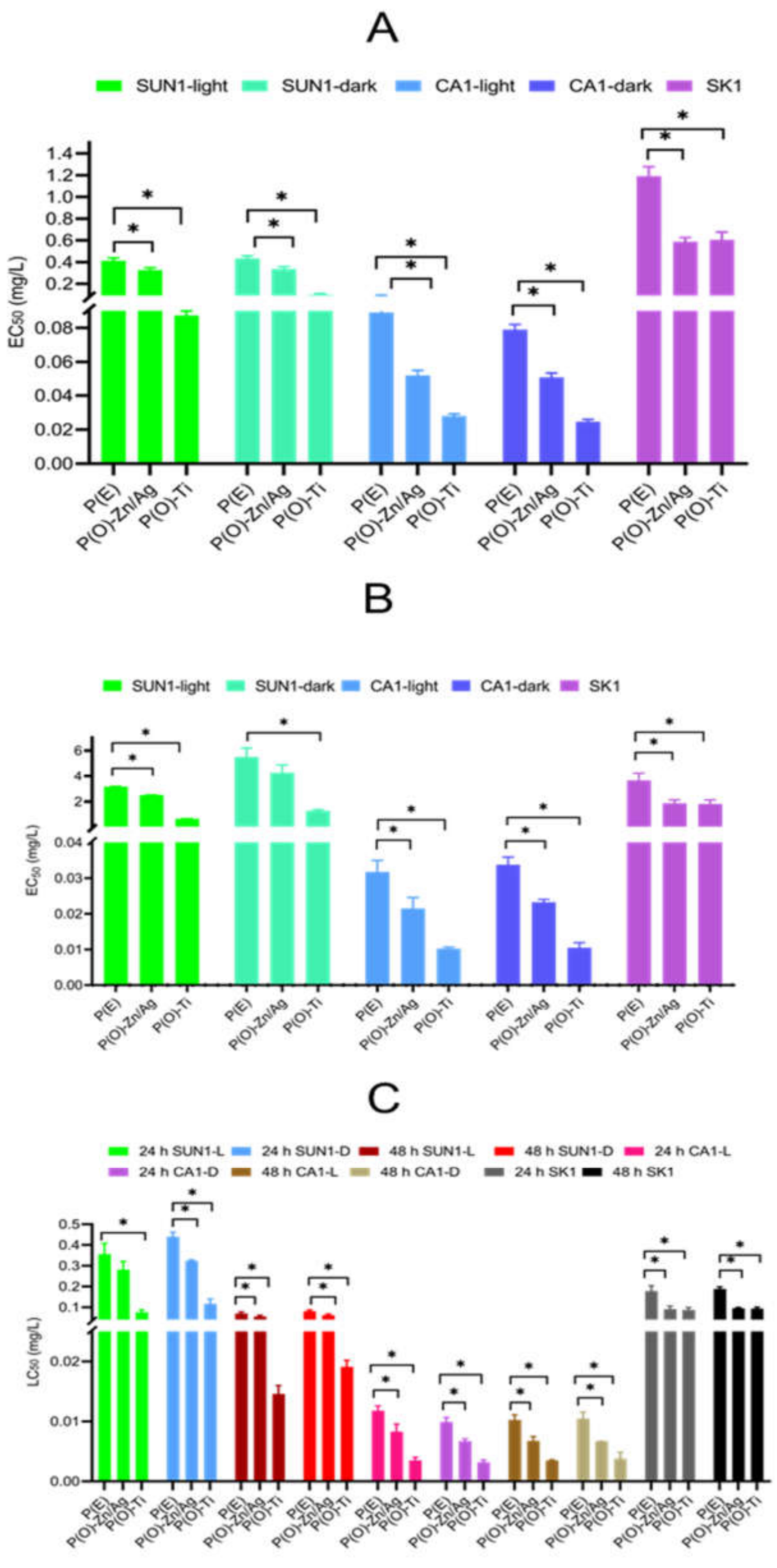

Figure S2. Comparison between theoretically expected [P(E)] and observed [P(O)] effects of SUN1, CA1 and SK1 PR-ENMs investigated on *P. subcapitata* (**A**), *S. polyrhiza* (**B**), and *D. magna* (**C**). * indicate statistical difference at *p*-value ≤ 0.05.

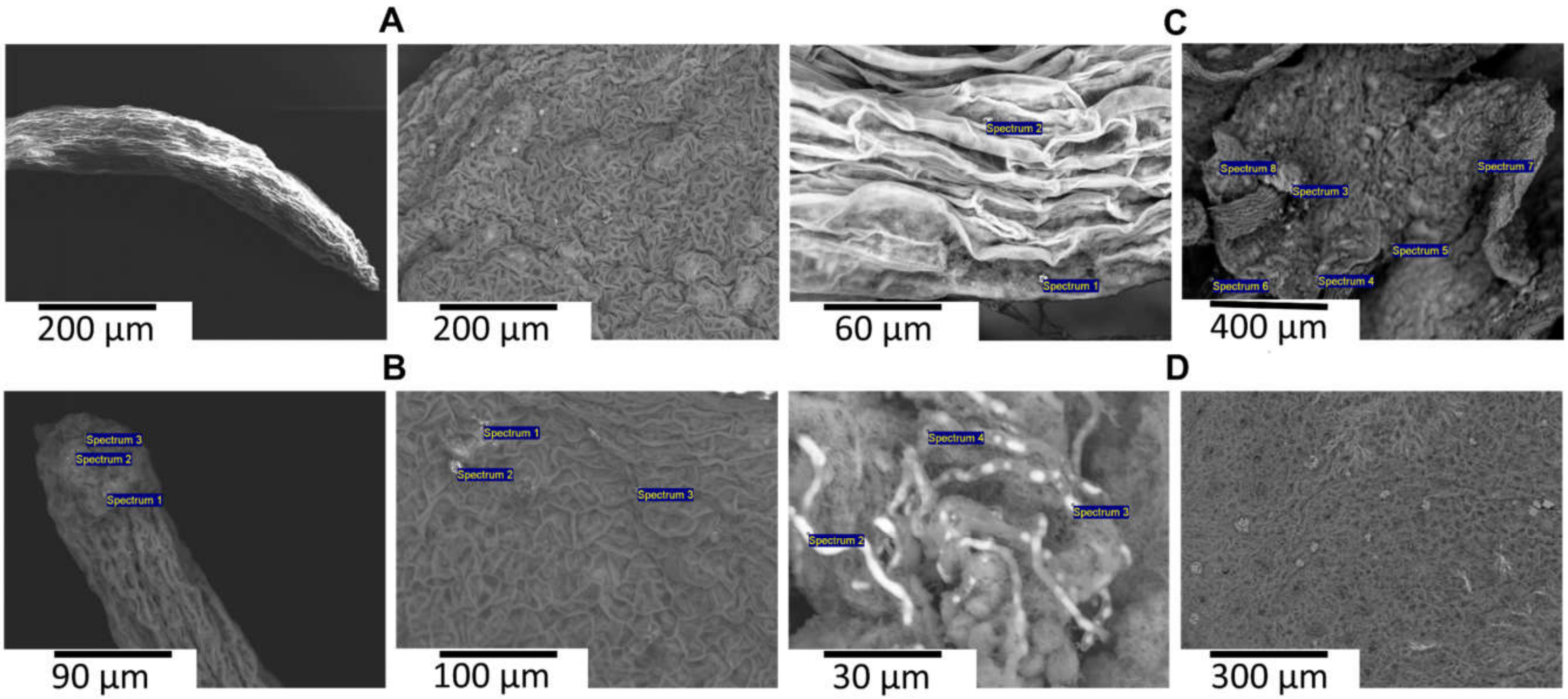

Figure S3. SEM images of the root (left) and frond (right) of *S. polyrhiza* control (**A**), *S. polyrhiza* exposed to 100 % (v/v) of SUN1 (**B**), SUN2 (**C**), and SUN3 (**D**) PR-ENMs; spectrum number points to the adsorbed SUNs' PR-ENMs.

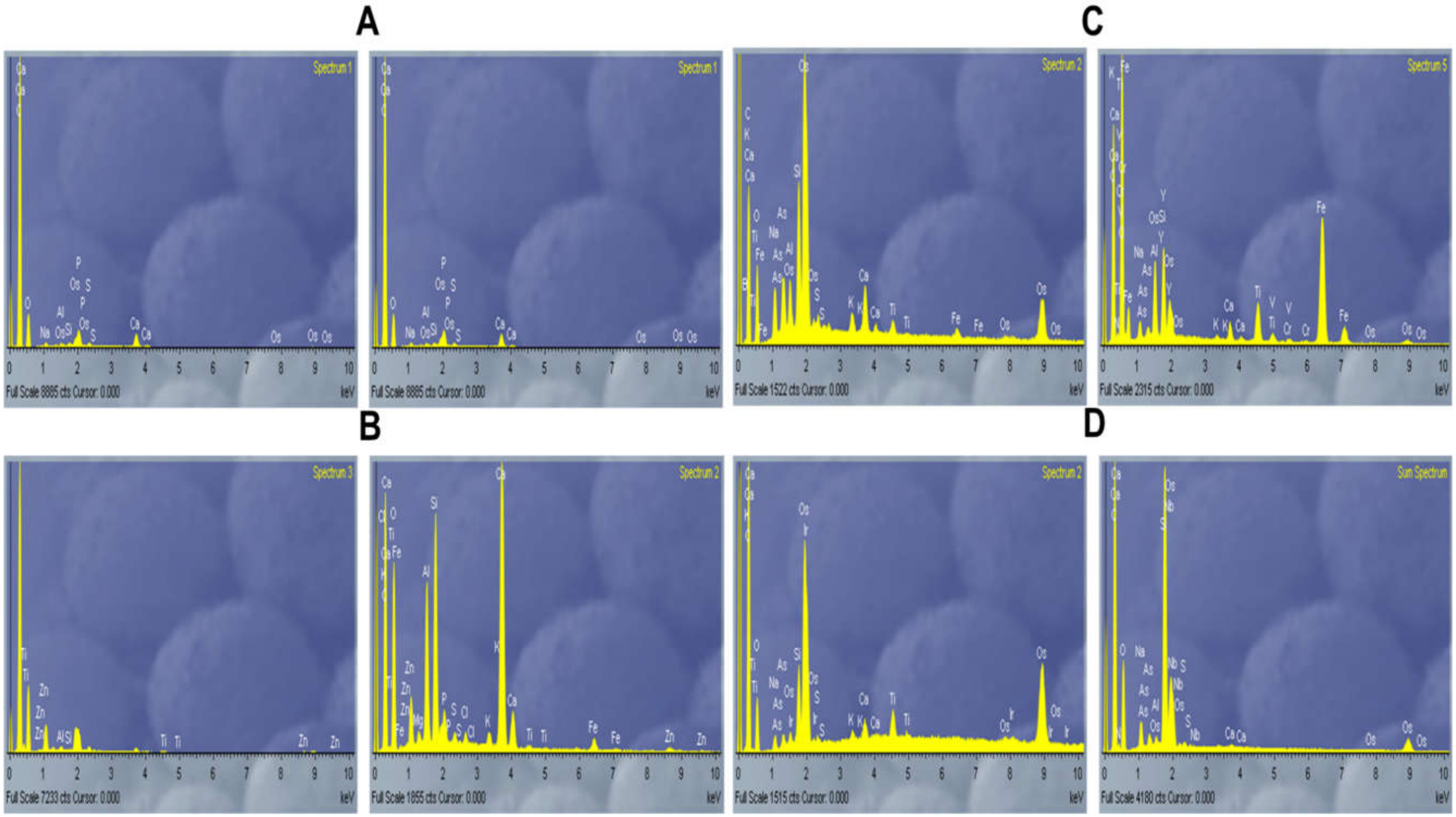

Figure S4. EDX images (corresponding to Figure 4) of the fronds (left) and roots (right) of *S. polyrhiza* control (**A**), *S. polyrhiza* exposed to 100% (v/v) SUN1 (**B**) and SUN3 (**C**) PR-ENMs.
